# Characteristics of soil moisture under different vegetation coverage in Horqin Sandy Land, northern China

**DOI:** 10.1371/journal.pone.0198805

**Published:** 2018-06-21

**Authors:** Tingting Yang, Musa Ala, Yushu Zhang, Jiabing Wu, Anzhi Wang, Dexin Guan

**Affiliations:** 1 Institute of Applied Ecology, Chinese Academy of Sciences, Shenyang, Liaoning, China; 2 University of Chinese Academy of Sciences, Beijing, China; 3 Institute of Atmospheric Environment, China Meteorological Administration, Shenyang, Liaoning, China; The University of Sydney, AUSTRALIA

## Abstract

Vegetation restoration as an effective sand fixation measure has made great achievements in China. However, soil water conditions deteriorate with the development and maturity of sand-fixing vegetation. In this study, we investigated the relationship between soil water content (SWC) and vegetation coverage (VC) at different portion (top, middle and bottom) on ten sand dunes during the growing season (April to October) in Horqin Sandy Land, northern China. We analyzed the temporal and spatial variation characteristics of SWC under different VC. The results indicate that VC and soil water storage were negatively correlated. The effect of vegetation on soil water storage on the sand dunes was greater in the dry season than the wet season. The VC and coefficient of coefficient of SWC were positively correlated at the 20 to 140 cm soil depth. As VC increased, the effect of drought stress increased at the 20 to 200 cm soil depths. The VC and SWC were negatively correlated at all the three portions of the sand dunes. According to soil water conditions and the concept of wilting humidity at different VC, we found that the suitable VC values were less than 0.46, 0.52, and 0.71 at the top, middle and bottom of the sand dunes, respectively.

## Introduction

Desertification has always been an important ecological problem in the arid and semi-arid regions of the world [1–3]. Large numbers of studies in in arid and semi-arid areas have demonstrated that vegetation is one of the most effective methods to reduce the hazards of desertification [1,3–6]. Water resources are the main limiting factor for vegetation growth in these areas [7–10]. Therefore, it is important to study the utilization and influence of vegetation on water. Plants affect the soil water balance in natural and constructed ecosystems by altering heat and moisture transfer from land surface to air [11]. Some studies have demonstrated that vegetation has a significant influence on temporal and spatial changes in soil moisture [12–16]. For example, English et al. [17] revealed that different grassland cover and species influenced soil moisture at different soil depths in a semi-arid environment. Vivoni et al. [18] found that vegetation affected plant interception and surface soil moisture. Wang et al. [10] showed that vegetation reduced the temporal stability of soil moisture. Acharya et al. [11] found that eastern red cedar (*Juniperus virginiana*) encroachment resulted in a more frequent depletion of soil water at the 80 cm soil depth. Özkan and Gökbulak [19] compared the effects of forest and herbaceous vegetation cover on soil moisture, and they reported that the removal of woody vegetation significantly increased the overall mean daily soil moisture of the soils from 32% to 48%.

China is one of the countries with the most severe desertification worldwide, and a large desertification control project has been carried out since 1950s [20–22]. Revegetation (such as trees, shrubs and herbs) is one of the most effective sand-fixing measures, and has made a great achievements in Horqin Sandy Land, northern China [23–25]. However, the desertification is aggravated owing to the vegetation coverage (VC) exceeds the bearing carry capacity of soil moisture [3,26,27]. Researchers have focused on the influence of vegetation on soil moisture and have found that different plant species can affect the temporal and spatial characteristics of the soil water content (SWC) [28,29]. In addition, the temporal and spatial characteristics of SWC are variable under different vegetation types [30–33]. Vegetation distribution also affects the spatial changes in SWC patterns [10,34,35]. These studies illustrated that vegetation can consume amounts of soil moisture and cause soil drying. Many scholars have proposed the principle of moderate VC for the management of sandy land. Yan et al. [36] studied the environmental impacts of shelter forests, and suggested that suitable tree species and plant density need to be considered. Yi et al. [37] studied the growth of *Pinus sylvestris* var. mongolica in response to density, and found a reasonable density of approximately 2100 stems per hectare at an age of approximately 20 years. Mo et al. [38] applied Eagleson’s ecohydrological optimality theory to determine the appropriate VC in vegetation restoration practice. These authors reported that the suitable VC (including grasslands/savanna, croplands and forests) is 0.318 in the Horqin sands. Musa et al. [29] found that the optimum density of *Caragana microphylla* was 2 m × 2 m in a windbreak and sand-fixing project. These studies indicated the necessity of suitable VC, and determined the suitable planting density of certain sandy species at a large scale. However, they did not study the relationship between the VC and soil moisture in sand dunes, and they didn’t define a threshold of suitable VC on sand dunes.

In this study, we conducted field sampling to measure the SWC at different sand dunes and to study the suitable VC in Horqin Sandy Land. It can make the construction of the vegetation more reasonable and effective. The goals of this study were to (a) analyze the temporal and spatial variation of soil moisture under different VC, (b) discuss the relationship between VC and SWC at the top, the middle, and the bottom portions of sand dunes and (c) identify the suitable VC on sand dunes. It is expected that these results can provide a theoretical basis for desertification control and vegetation construction.

## Materials and methods

### Study site

The study was conducted at Ulanaodu Experiment Station of Desertification (43°02′N, 119°39′E), Chinese Academy of Sciences, located in western Horqin Sandy Land, northeastern Inner Mongolia, China. The mean elevation is approximately 480 m a.s.l. The climate is a temperate, semiarid continental monsoon climate. Annual precipitation ranges from 200 mm to 300 mm, with 70% of the total falling with June to September. The mean annual open-pan evaporation is approximately 2000 mm. The mean annual temperature is 6.2°C with a minimum monthly mean temperature of -13.7°C in January and a maximum of 25.1°C in July. The relative humidity ranges from 50% to 60%. The mean annual wind velocity ranges from 3.2 to 4.1 m·s^-1^, and the prevailing wind direction is northwest in winter and spring but southwest to south in summer and autumn.

### Measurements

The study was conducted from April 21 to October 15, 2016. We selected ten sand dunes to measure the SWC and VC. On each sand dune, we set sampling points every 10 m from the top to the bottom of the sand dune. At each sampling point, soil samples were collected at 10 cm increments from the surface to a depth of 200 cm. The samples were collected at 10 to 15 d intervals. The SWC was measured by the oven drying method. We set up three 10 m×10 m sample areas at the top, middle and bottom of the sand dune and performed a detailed vegetation survey (length, width, height, branch diameter, number of branches) in August. The vegetation mainly included shrubs (*Caragana microphylla*), semi-shrubs (*Artemisia halodendron and Hedysarum fruticosum*) and herbaceous plants (*Setaria viridis and Pennisetum centrasiaticum*). The VC of the sand dunes was estimated (plant projection area divided by the sample area) in each plot [39–41].

The location and elevation of the sand dunes were measured with GPS. The GPS measurements usually have an instrumental error of the order of 2 to 5 m. Terrain information and VC of the sand dunes are shown in Table 1.

**Table 1:** Longitude, latitude, dominant species and VC of ten sand dunes.

DuneNumber	Longitude(N)	Latitude(W)	Dominant species	Vegetation coverage
No. 1	43°00'	119°38'	*Artemisia halodendron;*	0.046
No. 2	43°00'	119°38'	*Artemisia halodendron;*	0.061
No. 3	42°59'	119°37'	*Artemisia halodendron; Setaria viridis; Hedysarum fruticosum;*	0.15
No. 4	42°59'	119°39'	*Caragana microphylla;*	0.27
No. 5	42°59'	119°39'	*Caragana microphylla; Polygonum divaricatum;*	0.34
No. 6	43°00'	119°39'	*Caragana microphylla; Herba Artimisiae Sieversianae;*	0.39
No. 7	42°59'	119°39'	*Caragana microphylla; Herba Artimisiae Sieversianae;*	0.43
No. 8	42°59'	119°38'	*Caragana microphylla;Pennisetum centrasiaticum;*	0.45
No. 9	43°00'	119°38'	*Caragana microphylla; Hedysarum fruticosum;*	0.51
No. 10	42°59'	119°39'	*Caragana microphylla; Hedysarum fruticosum;*	0.62

### The characteristics of precipitation at the site

All meteorological data during the study experimental period (April 21 to October 15, 2016), including daily precipitation and daily evaporation, were obtained from an automated meteorological station (43°02' N, 119°65' E) located near the study site (< 1000 m,).

**Fig 1:**
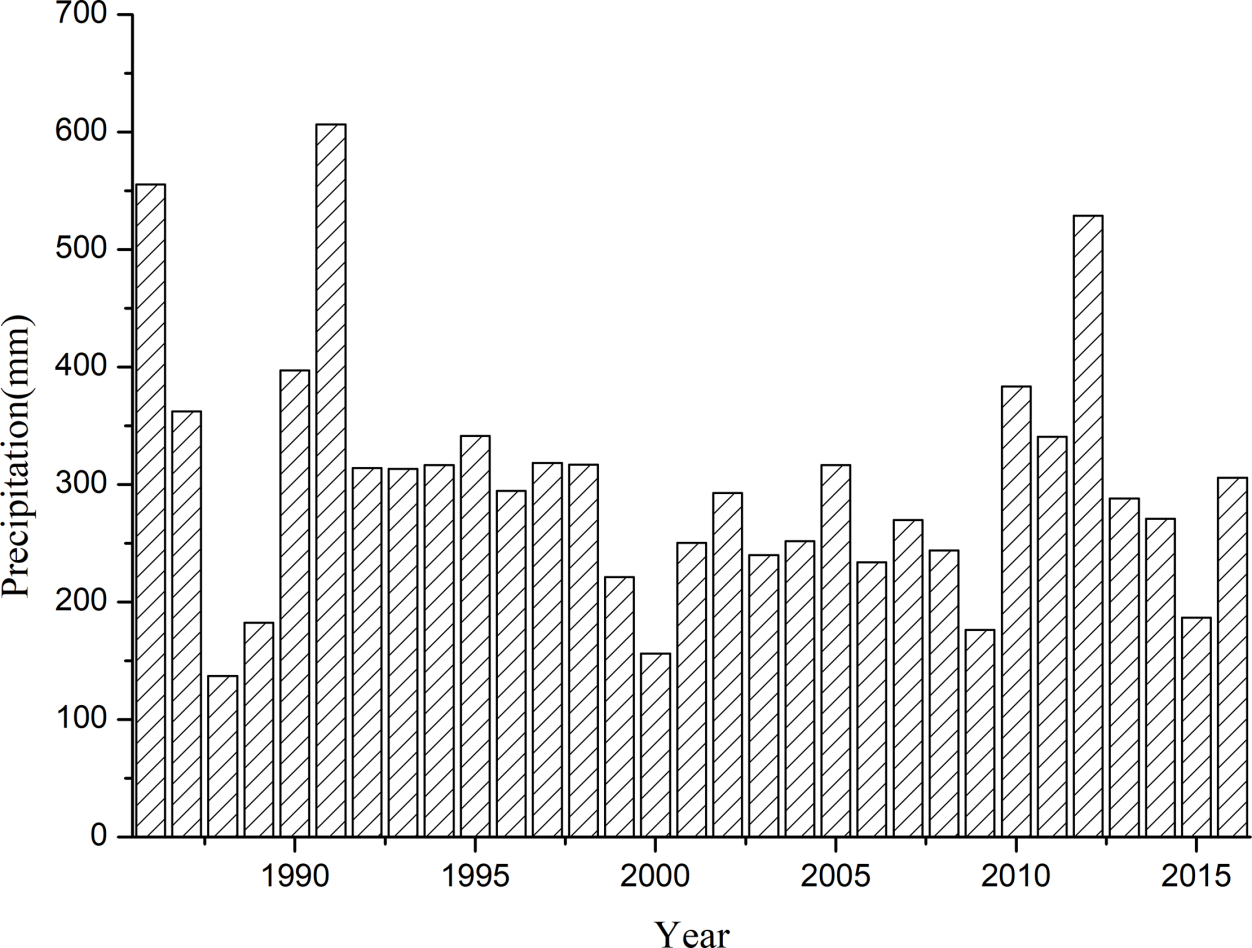
Annual precipitation at the study site for the recent 30 years (1986–2016).

**Fig 2:**
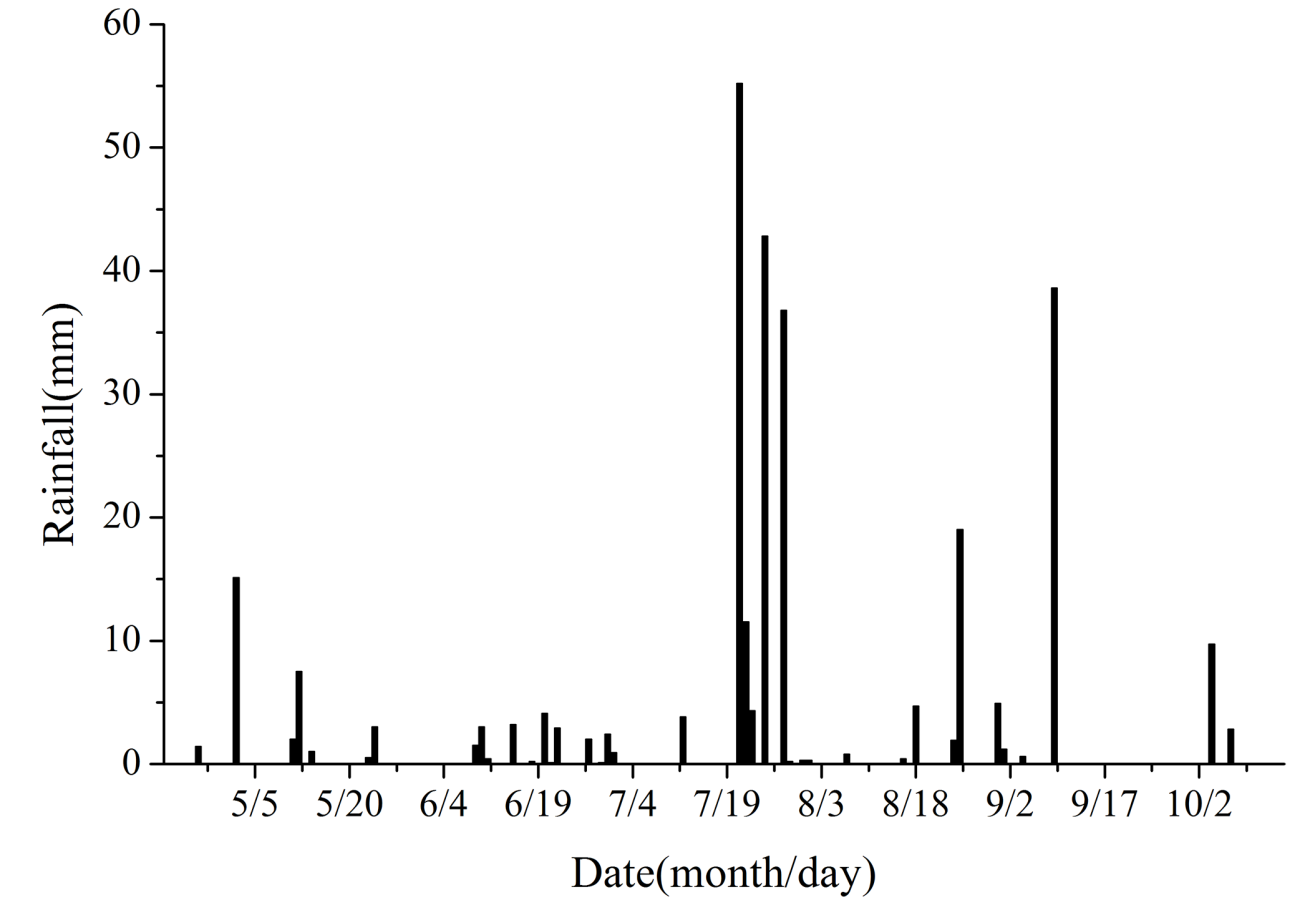
Rainfall during the experiment period (April 21, 2016 to October 15, 2016).

Fig 1 shows that the average precipitation in the recent 30 years was approximately 303.6 mm. The highest and lowest precipitation was 606.5 mm and 136.9 mm, which occurred in 1991 and 1988, respectively. Our experiment was carried out in 2016, and the annual rainfall was 305.6 mm. Therefore, the results of this study are likely to be applicable to normal precipitation conditions. Fig 2 shows that 40 rainfall events occurred during the experimental period, and the total rainfall was 291.1 mm. Among the rainfall events, 77.5% were < 5 mm. Effective precipitation event (≥ 5 mm) which means a direct influence on plant growth occurred nine times [42–44]. Among this nine events, five precipitation events occurred during July and August and these included relatively large rainfall amounts on July 21 (55.2 mm), July 25 (42.8 mm) and July 28 (36.8 mm). The seasonal distribution of rainfall is uneven and is mainly concentrated in July and August.

### Data analysis

In this study, we divided the sampling points into three portions, the top third of the sand dune was named “top”, the middle third was “middle” and the bottom third was “bottom”. The coefficient of coefficient is the ratio of the standard deviation to the variance; it is a measure of the degree of variation of the observed values in the data. Correlation analysis was performed using SPSS 19.0 to analyze the relationship between VC and soil water storage and relationship between VC and the SWC.

## Results

### Seasonal variation in soil water storage under different VC

**Fig 3:**
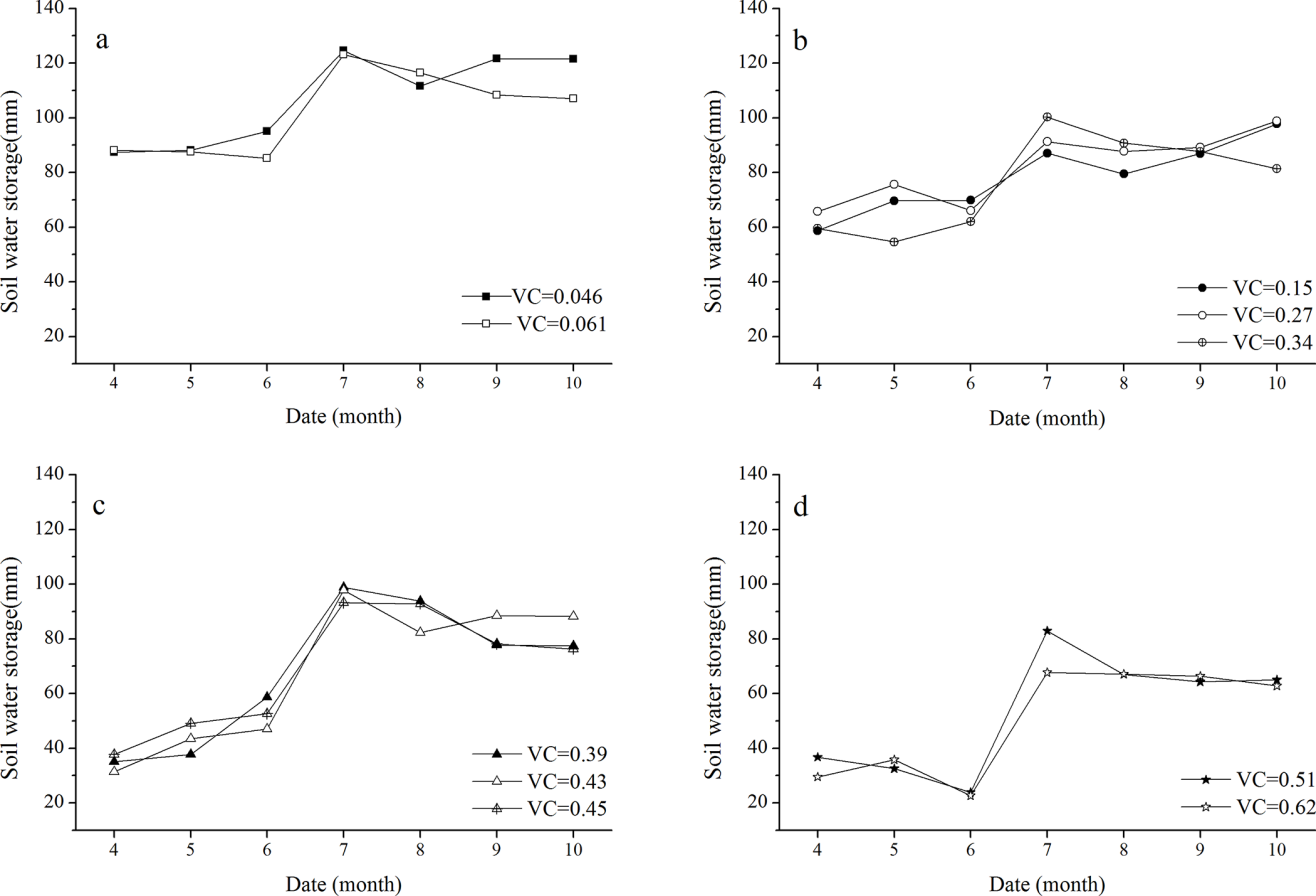
Seasonal variation in soil water storage (average value of all sampling points) at 0 to 200 cm soil depth on sand dunes with different VC. a: seasonal variation in soil water storage on sand dunes with group 1 (0.046 and 0.061); b: seasonal variation in soil water storage on sand dunes with group 2 (0.15, 0.27 and 0.34); c: seasonal variation in soil water storage on sand dunes with group 3 (0.39, 0.43, and 0.45); d: seasonal variation in soil water storage on sand dunes with group 4 (0.51 and 0.62).

Fig 3 shows the monthly variation in soil water storage (average value of all sampling points) at 0 to 200 cm soil depths under different VC. We divided the 10 dunes into four groups based on VC values (group 1: 0.046 and 0.061; group 2: 0.15, 0.27 and 0.34; group 3: 0.39, 0.43 and 0.45; group 4: 0.51 and 0.62). The soil water storage showed a similar seasonal variation trend in the four groups. The storage in July was the highest during the growing season. We assigned April to June as the dry season and July to October as the wet season based on monthly rainfall. The average soil water storage of all dunes was 56.2 mm and 90.0 mm in the dry and wet seasons respectively, showing that soil water storage in the wet season was significantly higher than that in the dry season.

There are distinct differences in soil water storage under different VC. The mean soil water storage were 102.6, 80.0, 68.6 and 51.0 mm in groups 1–4, respectively, during the growing season. The soil water storage was > 85 mm in group 1 and < 85 mm in group 4 during the entire growing season (Fig 3A and 3D). The average soil water storage exhibited a small difference between group 2 and group 3 in the wet season; the difference between the two was only 7 mm (Fig 3B and 3C)). However, the result of compared the average soil water storage of group 2 with group 3 in the dry season indicated a large difference between the two groups, i.e., values of 65 mm and 44 mm, respectively, with a difference of 21 mm. These results show that the influence of VC on soil water storage was more remarkable in the dry season. Moreover, the seasonal variation in soil moisture was mainly affected by the seasonal distribution of rainfall. Therefore, it is indirectly concluded that the soil water storage is affected by rainfall.

**Fig 4:**
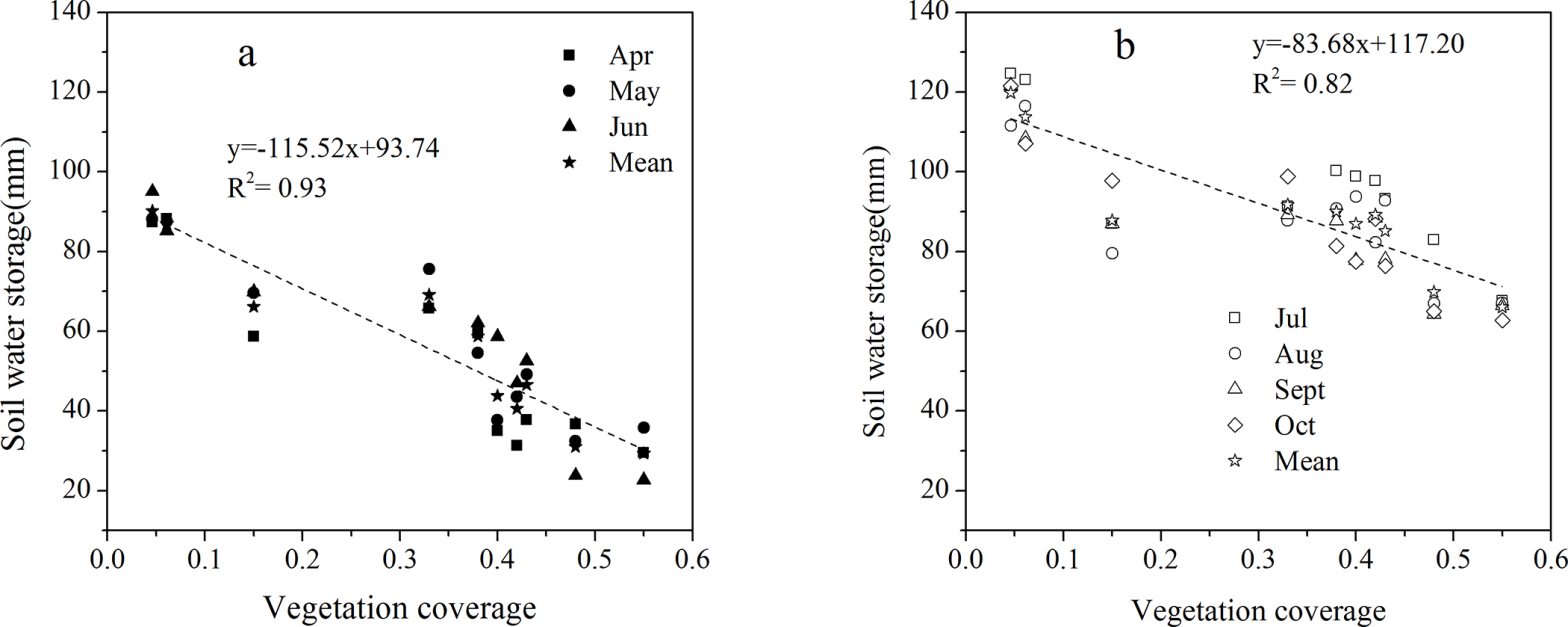
Correlations between VC and soil water storage in the dry (a) and wet season (b).

Fig 4 shows the regression of VC and soil water storage in the dry and wet seasons respectively. It can be see that soil water storage and VC were negatively correlated, and the R^2^ value were > 80% (p<0.01) in the dry and wet seasons. This shows that soil moisture is inversely proportional to VC. The results indicated that soil water storage decreased with the increase of VC.

### Vertical change in SWC under different VC

**Fig 5:**
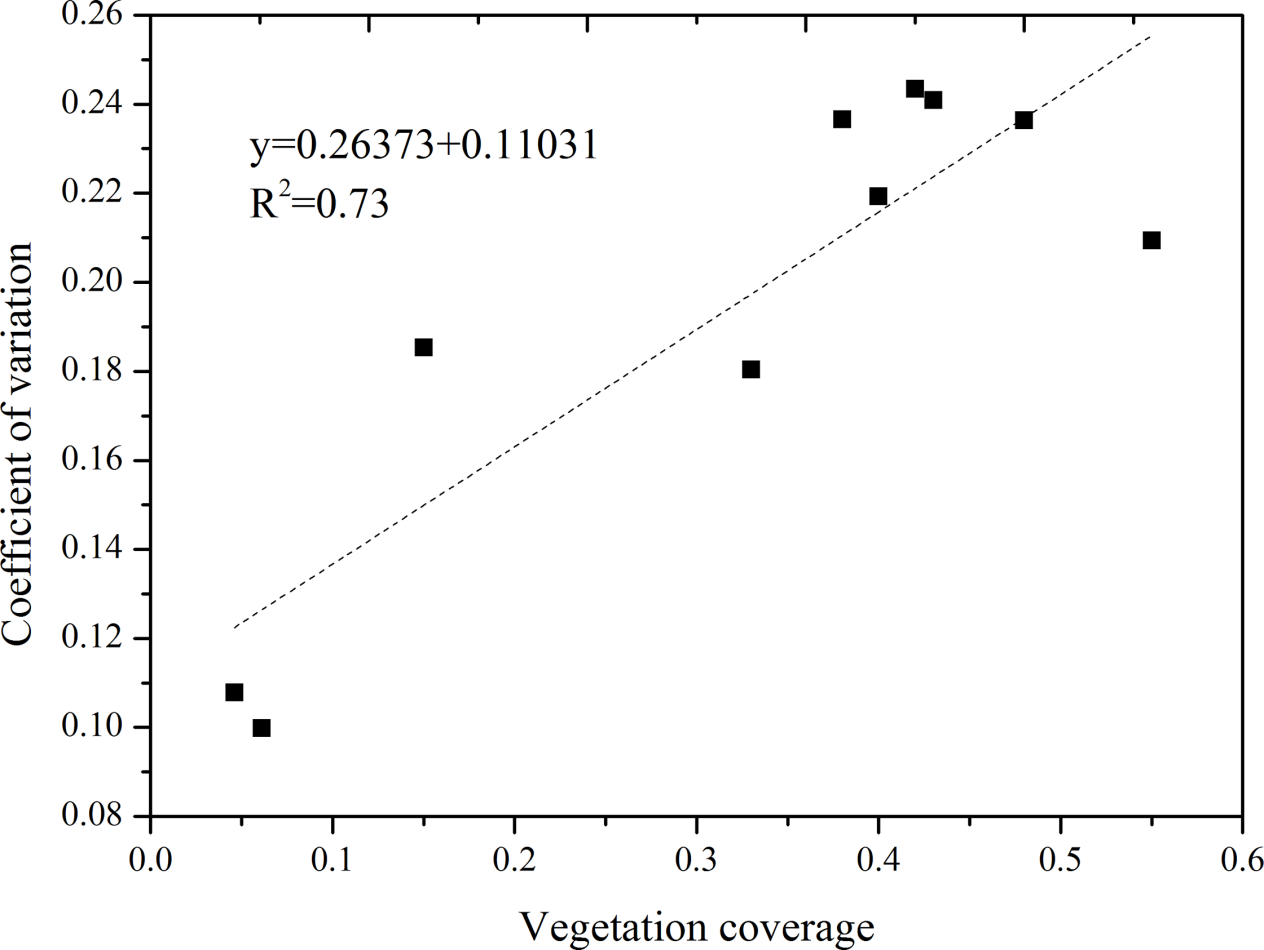
Correlations between VC and the coefficient of coefficient at 20 to 140 cm soil depth during the growing season.

We divided the soil into three layers (0 to 20, 20 to 140 and 140 to 200 cm) according to the vertical variation characteristics of the SWC. At 0 to 20 cm, the SWC was low (mean SWC = 1.85% on ten sand dunes) during the entire growth season. The maximum value (2.4%) occurred in the dune with VC = 0.046, and the minimum value (1.3%) occurred in the dune with VC = 0.62. And the fluctuation of SWC was greatest at 0 to 20 cm soil depth, and the average coefficient of coefficient exceeded 0.56 of the ten sand dunes. The average coefficient of coefficient of SWC was lowest in July for all dunes, which indicated that the SWC of surface soil was mainly affected by rainfall. Fig 5 shows correlations between VC and the coefficient of coefficient of the vertical SWC at 20 to 140 cm soil depth during the growing season. VC and the coefficient of coefficient were positively correlated; the R^2^ value > 0.7 (p<0.01). As VC increased, there was a greater fluctuation in SWC at 20 to 140 cm soil depths. The SWC was mainly affected by root water absorption at the 20 to 140 cm. At a soil depth of 140 to 200 cm, the SWC was high and relatively stable during the entire growing season (mean value of all dunes was 3%), and the mean coefficient of coefficient was less than 0.21 for the ten sand dunes.

**Fig 6:**
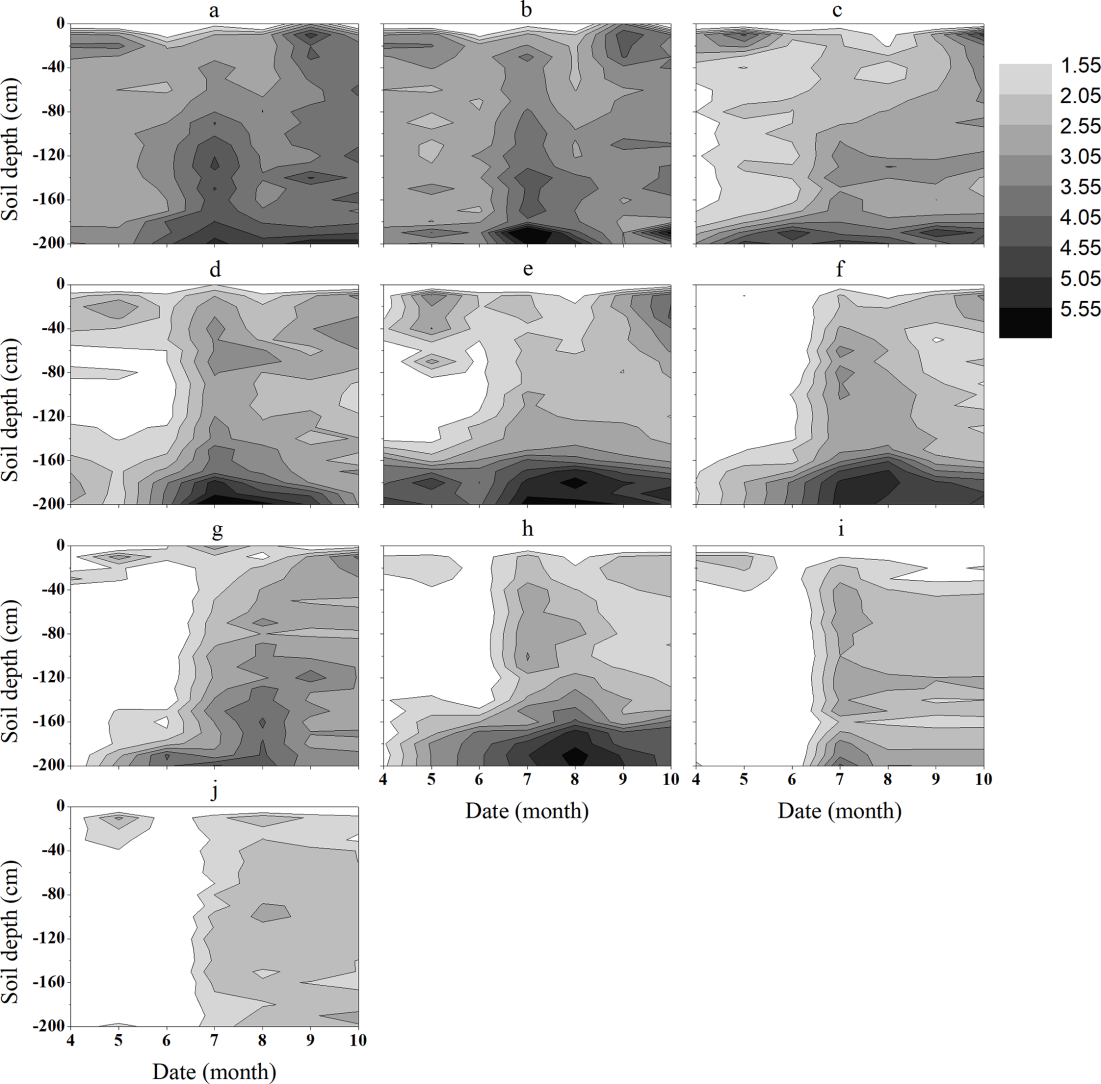
Variation in the SWC (mean value of all sampling points) on vertical profile (0 to 200 cm soil depth) in sand dunes with different VC values (a: VC = 0.046; b: VC = 0.061; c: VC = 0.15; d: VC = 0.27; e: VC = 0.34; f: VC = 0.39; g: VC = 0.43; h: VC = 0.45; i: VC = 0.51; j: VC = 0.62).

*Caragana microphylla* Lam. (Fabaceae) was the most drought-resistant plant in our study area. We assigned the wilting humidity value (1.55%) of *C*. *microphylla* as the standard. When SWC is < 1.55%, plants are under drought stress [45,46]. Fig 6 shows the variation of SWC (mean value of all sampling points) in the vertical soil profile during the growing season on sand dunes with different VC. The white areas represent the soil under drought stress. For the dunes with VC is 0.046 and 0.061, there was no drought stress in the soil depth range of 20 to 200 cm during the entire growing season. As VC increased, the range of soil under drought stress increased gradually, and the duration was prolonged. When the VC was 0.48 and 0.55, the soil profile under drought stress reached 200 cm in the dry season. This indicated that the vertical profile of the SWC was affected by VC, i.e., high VC leads to soil water loss and makes the dune less suitable for the growth of vegetation.

### Relationship between VC and SWC

**Fig 7:**
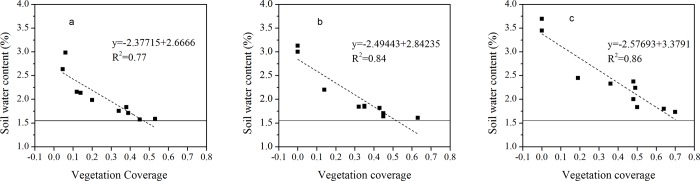
Correlations between VC and the SWC at three portions on sand dunes during the growing season. SWC of 1.55% was used as the evaluation criterion of drought stress. a: correlations between VC and the SWC at the top of the sand dunes; b: correlations between VC and the SWC in the middle of the sand dunes; c: correlations between VC and the SWC at the bottom of the sand dunes.

Fig 7 shows the correlation between VC and the SWC at the three portions of the sand dunes. The relationship between VC and the SWC in the three portions indicated the same trend, i.e., that VC and the SWC are negatively correlated (p<0.01). These results demonstrated that the SWC decreased with the increase of VC in three portions of sand dunes. It can be seen that high VC values can lead to soil drought, and this is detrimental to vegetation growth. We established the wilting humidity value (1.55%) as the standard of soil drought, and took the intersection of wilting humidity value with the trend line extension as a threshold of suitable VC. When the VC was lower than the threshold, we regarded it as suitable VC. In summary, at the top, middle and bottom of the sand dunes, suitable VC was less than 0.46, 0.52, and 0.71, respectively.

## Discussion

### The temporal variation characteristics of soil moisture under different VC

Soil moisture has obvious temporal variation in arid and semi-arid areas. Zhao et al. [47] studied the temporal variation in soil moisture at different geomorphic sites, and they found that March and April were the periods of precipitation recharge, whereas May and June were moisture depletion periods. Li et al. [48] studied the temporal variation in soil moisture under four plant communities in the Otindag Sandy Land. It was found that the SWC could be divided into three periods according to its seasonal variation. Precipitation storage occurred in April and May, water was consumed from June to mid-September, and there was a slow recovery of water from late September to October. Cui et al. [49] analyzed the seasonal variation in soil moisture under different sand dune types in the Mu Su Sandy Land. They found that the SWC of fixed sandy land was lowest in summer. And the SWC of semi-fixed and moving sandy land were lowest in spring. Liu et al. [50] discovered that the soil moisture under three vegetation types (herbs, shrubs, and bare sand) exhibited obvious difference between dry and wet seasonal in the aspect of variation in the Horqin sandy land. These studies showed a clear seasonal variation in the SWC in arid and semi-arid areas and indicated that rainfall is the main source of soil water replenishment [51–54]. There are fewer reports concerning the temporal variation in soil moisture under different VC. In our study, we found that soil water storage and VC were negatively correlated in the dry (y = -115.52x+93.74) and wet (y = -83.68x+117.20) seasons. With increasing VC, the soil water storage showed an obvious decreasing trend in both the dry and wet seasons. The results indicated that VC affected the variation in soil water storage, and the influence of VC in the dry season was more obvious.

### Vertical variation characteristics of soil moisture under different VC

Numerous studies found that the vertical variability of soil moisture under different vegetation cover was different in the Horqin Sandy Land. For example, Lin et al. [55] studied the change of SWC in the vertical soil profile under three different types of plant barriers. The study showed a large fluctuation at the 20 to 40 cm soil layer of the three plant barriers. And the change of SWC was different at different depths (10 to 20, 20 to 60, 60 to 80 and 80 to 100 cm) in the three plant barriers. Niu et al. [56] analyzed the spatial variation in soil moisture under different land uses in Horqin Sandy Land. They found that the SWC of surface (0 to 20 cm) was affected by rainfall, and it fluctuated considerably. Influenced by evaporation, the soil moisture in the rooting zone (0 to 80 cm) was highest in grassland but lowest beneath shrubs among five land use patterns. The SWC in the deep soil layer (80 to 120 cm) of grassland and inter-dunes was remarkably higher than it of the other land use patterns, whereas the lowest SWC was appeared beneath shrubs. Yan-Qing et al. [57] analyzed the change of soil moisture in different types of sandy land in Horqin Sandy Land. The results showed that the SWC was increased with the increasing of depth in the 0 to 40 cm soil layer in the three types of sandy land. But it was decreased with the increasing of depth in the 0 to 130 cm soil layer. At the 131 to 160 cm soil depth, the SWC decreased continuously in mobile dunes, but it increased in fixed dunes and sandy grasslands. These results show that different vegetation cover have different effects on the vertical variation of the SWC. Generally, the soil can be divided into three layers according to the variation characteristics of the SWC. The SWC is affected by precipitation and evaporation at 0 to 20 cm soil depth, and the fluctuations are significant. The SWC is affected by root water absorption at 20 to 140 cm soil depth and it is relatively stable at this depth. And the SWC is high at 140 to 200 cm soil depth. [34,58–60]. Our study showed the same variability in the SWC and also found that VC is directly proportional to the coefficient of coefficient of the SWC. However, previous studies merely compared and analyzed the changes in the SWC among different land use types and among different types of sandy land across the entire vertical soil profile. None of the above studies involved the soil moisture changes under different VC, whereas we analyzed the change of SWC in the entire vertical soil profile under different VC. We found that different VC affect the SWC at different depths in this study. The SWC was high when the VC was 0.046 and 0.061 during the growing season at 20 to 200 cm soil depth. The main reason for this phenomenon is that the sandy soil has faster soil infiltration, resulting in a dry sand layer that inhibits soil evaporation. In addition, the water consumption of vegetation is very low [4,57,60]. When the VC was 0.48 and 0.55, the soil was under drought stress at 20 to 200 cm soil depth in the dry season. As the VC increased, the soil (specifically, the depth of soil) under drought stress increased gradually and the drought duration was prolonged. The main reason is that as VC increasing, the transpiration will consume a large amount of water [61,62].

### Relationship between VC and SWC at different portions on the sand dunes

The relationship between VC and SWC is controversial. Cui et al. [49] analyzed the characteristics of SWC under different VC (>40%, 20% - 30% and <5%). The results showed that the SWC at 40% coverage was lowest. Zhang et al. [63] analyzed the relationship between the coverage and SWC of an *Artemisia ordosica* community. The results showed that *A*. *ordosica* communities with different coverage effected the SWC at different soil depths. The SWC in the 0 to 20 cm soil layer increased obviously with the increase in community coverage, but it decreased with the increase in community coverage at a depth of 20 to 80 cm. Ala et al. [45] studied the relationship between the suitable density of *Caragana microphylla* and soil moisture. They found that the SWC was lower than 1.55% under 0.5 m×1 m and 1 m×2 m straw checkerboard sand barriers but it was consistently maintained at a level higher than 1.6% during the growth season under 2 m × 2 m densities. These findings suggested that the increasing of VC will lead to a decrease of SWC. Our study also found that with the increasing of VC, the SWC showed a downward trend. The reason is that high VC reduces rainfall recharge to the soil through canopy interception and has high root water consumption [58,64]. However, these studies only contrast the relationships between different VC and SWC. They did not specifically study the relationship between VC and the SWC at different portions of sand dunes. We found that the SWC had a slightly difference between the top and the middle of sand dune, but the SWC of both of these portions was significantly lower than that at the bottom of the sand dune. In addition, there were differences between VC and the SWC at different portions of dunes at the 0 to 140 cm soil depth. Based on the relationship between VC and the SWC in the three portions of the sand dunes, we determined a suitable VC. At the top, middle and bottom of sand dunes, the suitable VC is less than 0.46, 0.52, and 0.71, respectively. These results provide a basis for optimal vegetation restoration.

## Conclusion

Based on the analysis of the relationship between VC and soil water storage, we found that the soil water storage decreased with the increasing of VC, and in the dry season, the influence of VC was more obviously than that of wet season. The SWC in the 20 to 140 cm soil layer was affected by the VC most strongly, and the coefficient of coefficient of the soil was proportional to the VC. In the 20 to 200 cm soil profile, the extent of soil under drought stress showed an increasing trend in response to increased VC. The optimum VC of the three portions was less than 0.46 (top), 0.52 (middle) or 0.71 (bottom). However, different vegetation communities may affect the relationship between VC and SWC through vegetation water consumption and root distribution [65–67]. The potential interference needs to be further discussed in the future.
